# Mediterranean precipitation isoscape preserved in bone collagen δ^2^H

**DOI:** 10.1038/s41598-020-65407-0

**Published:** 2020-05-22

**Authors:** Linda M. Reynard, Saskia E. Ryan, Michele Guirguis, Miguel Contreras-Martínez, Elisa Pompianu, Damià Ramis, Peter van Dommelen, Noreen Tuross

**Affiliations:** 1 Department of Human Evolutionary Biology, Harvard University, 11 Divinity Avenue, Cambridge, MA USA; 2 Università di Sassari, Dipartimento di Storia, Scienze dell’Uomo e della Formazione, Sassari, Sardinia Italy; 3 Museo Arqueologico Regional de Madrid, Pza. de las Bernardas s/n, Alcalá De Henares, Spain; 4 Independent researcher, via Oristano 116, 09094 Marrubiu (Or), Sardinia Italy; 5 Independent researcher, Cl Moragues 34, 07006 Palma de Mallorca, Balearic Islands Spain; 6 Joukowsky Institute for Archaeology and the Ancient World, Brown University, Providence, RI USA; 7 Present Address: Archéozoologie, Archéobotanique: Sociétés, Pratiques et Environnements (AASPE, UMR 7209), Muséum national d’Histoire naturelle, CNRS, CP56, 55 rue Buffon, 75005 Paris, France

**Keywords:** Geochemistry, Stable isotope analysis

## Abstract

The prehistory of the Mediterranean region has long been a subject of considerable interest, particularly the links between human groups and regions of origin. We utilize the spatial variation in the δ^2^H and δ^18^O values of precipitation (isoscapes) to develop proxies for geographic locations of fauna and humans. Bone collagen hydrogen isotope ratios (δ^2^H) in cattle (and to a lesser extent, ovicaprids) across the Mediterranean reflect the isotopic differences observed in rainfall (but δ^18^O values do not). We conclude that δ^2^H in herbivore bone collagen can be used as a geolocation tracer and for palaeoenvironmental studies such as tracing past isotopic variations in the global hydrological cycle. In contrast, human bone δ^2^H values are relatively tightly grouped and highly distinct from precipitation δ^2^H values, likely due to human-specific food practices and environmental modifications. Given the inter-species variability in δ^2^H, care should be taken in the species selected for study.

## Introduction

Migration is a profoundly important part of human existence, from the dispersal of *Homo sapiens* out of Africa to the past and present movement of humans over the globe. The new tools available in the biological and physical sciences have contributed to a lively interest in migration studies of past populations and individuals. The scale at which migration can be studied is quite varied. For ancient DNA, continental and global-scale studies have examined the genomic fine structure of populations and movement or inter-mixing between them^1^. However, ancient DNA studies lack power to resolve the timing and duration of some migration(s). Separately, variation in stable isotope ratios of tissues can be used to identify migrating individuals or groups directly.

The Mediterranean region has a long history of exchanges and migration, which substantially intensified from the first millennium BCE^2,3^. We focus here on the Bronze and Iron Ages and the Phoenician-Punic periods (ca. 3000–500 BCE) – a time of increasing social complexity and pan-Mediterranean linkages and exchange. Until now it has been difficult to use stable isotopes to identify migration in this region. In general, latitude and altitude changes can be useful in tracking migrations due to the environmental and dietary differences that often accompany these trajectories. In contrast, the variation in carbon isotopes (δ^13^C) through broad regions of the latitudinally similar Mediterranean are very slight and trend predominantly along a north-south gradient in Europe, making migrations around the Mediterranean invisible to this biomarker^4^. Nitrogen isotopes (δ^15^N) are related to soil composition and environment at a very local scale and are not useful for geolocation^5^. No comprehensive isoscape (isotopic landscape) of bio-available strontium isotope (^87^Sr/^86^Sr) values covering the entire Mediterranean region has been established. Given the large spatial extent of the region, overlapping Sr isotope values of geographically distant regions are found^6^, rendering this approach an inadequate tool when used alone for pan-Mediterranean migration studies. Instead, we investigate variations in hydrogen (δ^2^H) and oxygen (δ^18^O) isotopes in bone collagen of fauna and humans and their relationship with a well-developed isoscape across the region based on precipitation δ^2^H and δ^18^O.

Hydrogen and oxygen isotope ratio variations are products of the global hydrological cycle, influenced by temperature and atmospheric transport from source to precipitation location; as a result, δ^2^H and δ^18^O in precipitation can vary significantly with geography. The distribution of these isotopes across the Mediterranean basin was first described by Gat and Carmi^7^. Globally, δ^2^H and δ^18^O in precipitation co-vary, forming the Global Meteoric Water Line (GMWL)^8^. Deuterium excess (*d*), defined as *d* = δ^2^H – 8 δ^18^O, varies across the globe and is influenced by local aridity and the relative humidity of originating air masses^9^. The isotopic variation in precipitation can be incorporated into biological tissues and serve as a tracer of location, as exemplified by δ^2^H in bird feather keratin used for geo-location of the origin of migrant birds^10,11^, δ^2^H and δ^18^O variation in human hair keratin with water variation^12^, and δ^18^O in tooth enamel apatites used to distinguish non-local individuals^13^. Herding strategies and vegetation variation have also been investigated with time-resolved δ^18^O values of tooth enamel^14^. In contrast, organic H and organic O in bone collagen have not been developed as a geo-location tool, with the exception of an early study on deer^15^. Feeding and observational studies show that ingested water isotopic composition is incorporated in bone collagen H and O^16–18^, and ovicaprid dentin collagen^19^, and thus bone collagen δ^2^H and δ^18^O should reflect the environmental source water and the origin of the individual.

For applications to palaeoenvironmental and past migration questions, bone collagen offers advantages over other tissues: i) bones are often preserved in the geological and archaeological record (in contrast to keratin), allowing direct isotopic study of the individual of interest; ii) bone collagen reflects an integrated, albeit variable time period^20,21^ in contrast to enamel apatite, which is formed at discrete ages and may not capture migration ‘events’; iii) collagen may be directly dated by radiocarbon. Further, ideal sources of δ^2^H and δ^18^O of the past, waters and plants, are largely unavailable due to lack of preservation, so that bone collagen may be useful in recording and tracing past environmental variation^22^.

**Figure 1:**
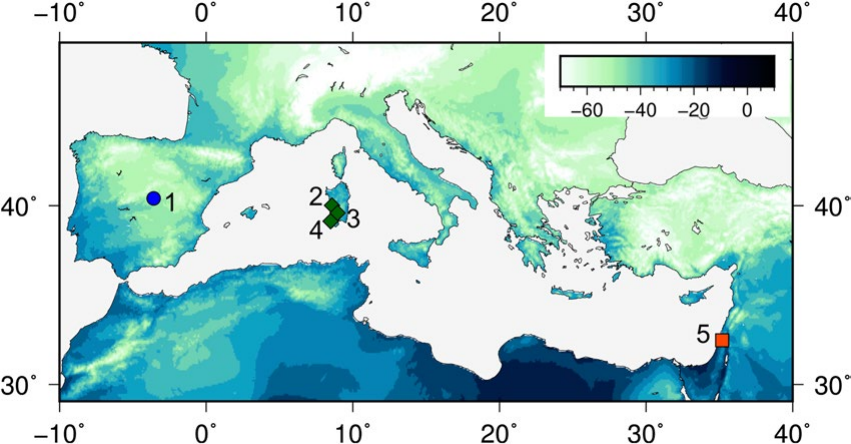
Mean annual precipitation δ^2^H (‰). Sample sites are Los Berrocales (1), S’Urachi (2), Villamar (3), Monte Sirai (4), Megiddo (5). Published gridded data at 5’ resolution are used; these data use the GNIP data set, plus latitude and altitude to generate a temperature-related rainout isotopic estimate which is used to generate a best-fit model^38–40^. The map was created using Generic Mapping Tools version 5.3.3^41,42^.

Here, we present sub-fossil bone collagen δ^2^H and δ^18^O from a range of archaeological sites spanning 3500 km across the Mediterranean basin (Fig. 1). Precipitation δ^2^H, *d* excess, and to a lesser degree δ^18^O vary substantially from west (lowest values) to east (highest values), with δ^2^H ranging over 25‰, δ^18^O over 2‰, and *d* excess over 9‰ from Spain to Israel (Fig. 1, Table S1). We investigate whether bone collagen δ^2^H and δ^18^O values vary spatially, in concert with the longitudinal variation in precipitation δ^2^H, δ^18^O, and *d* excess values.

## Results and Discussion

**Figure 2:**
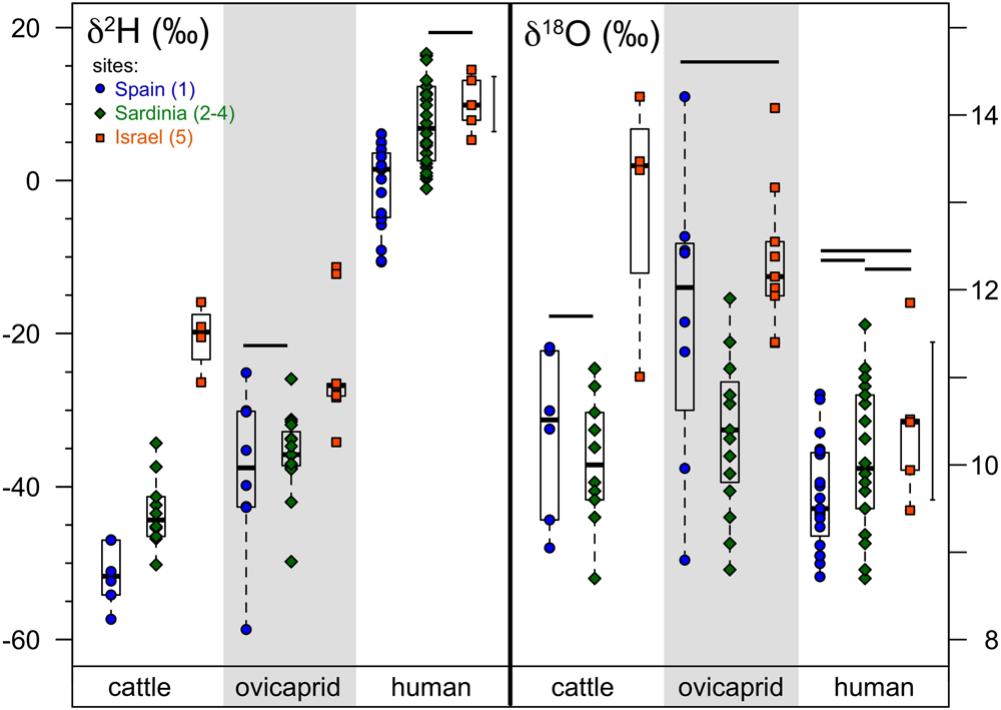
Collagen δ^2^H (‰) and δ^18^O (‰) by species and site, with numbers corresponding to site locations in Fig. 1, with sites Los Berrocales in Spain (1) and Megiddo in Israel (5). The error bar corresponds to our laboratory’s long term reproducibility (1 sd of long term mean, n = 80 for δ^2^H, n = 161 for δ^18^O). Horizontal bars indicate no significant differences between groups (p > 0.05, Tukey’s Honest Significant Differences, Table S2); there are fewer significant differences between groups in δ^18^O values than δ^2^H values. Cattle δ^2^H are significantly different by site for all three pairwise comparisons (Table S2).

Bone collagen δ^2^H and δ^18^O were measured across 5 archaeological sites (Figs. 1–2, Tables S4-S6). δ^2^H values in cattle collagen are lowest at the westernmost site (1, Los Berrocales), higher at the central site (2, Sardinia), and highest at the easternmost site (5, Megiddo), and ovicaprid δ^2^H values are lower in the centre/west than the easternmost site (Fig. 2), matching the isotopic pattern in precipitation. Collagen δ^2^H in cattle is significantly correlated with mean precipitation δ^2^H (p = 0.001, r^2^ = 0.46, slope=0.75 ± 0.19, 1 se, Table S3) and *d* excess (p = 1.2 × 10^–8^, r^2^ = 0.84, Table S3). Ovicaprids also show a significant but weaker correlation between collagen δ^2^H and precipitation δ^2^H and *d* (Table S3). Given *d* is a parameter related to precipitation δ^2^H (*d* = δ^2^H – 8 δ^18^O), the correlation of collagen δ^2^H and rainfall *d* values is consistent. The bone collagen of these animals is incorporating the isotopic variation in precipitation across the Mediterranean basin, both directly from water and through food sources. The faunal δ^2^H data are consistent with the modern pattern of precipitation, with lower δ^2^H and *d* excess, increased rainfall, and lower aridity in the west compared to the east (Fig. 2, Table S1). The climate in the Mediterranean region in the late Holocene was generally stable after mid-Holocene aridification (see Supplementary Information). If there were any shift in the isotopic composition of precipitation between ~3000–4000 years ago and the present, it has not obscured this collagen-precipitation relationship. In contrast, neither cattle nor ovicaprid collagen δ^18^O is correlated with mean precipitation δ^18^O (p = 0.09–0.90, r^2^ = 0.00–0.09, Table S3).

The covariation of δ^2^H in collagen and precipitation is consistent with the incorporation of H from drinking water. Additionally, food hydrogen (plant material) and plant water hydrogen also contribute to H in collagen^16,17^. If the isotopic fractionation from precipitation to plant water and/or plant tissue is roughly similar across the environments sampled here, the same isotopic offset should be translated into the herbivore bone collagen via food and plant water inputs. The large shift in the absolute values of δ^2^H and δ^18^O in collagen vs. meteoric water indicates that there is significant fractionation from water to animal tissue (or from water to plant to animal tissue) as noted in previous studies^16,17^, but despite this, the resulting bone collagen δ^2^H maintains a relationship with precipitation δ^2^H and *d* excess values.

The stronger relationship between bone collagen δ^2^H and precipitation δ^2^H in cattle than in ovicaprids may be due to differences in water and plant consumption. Water flux scales with body size^23,24^ and larger-bodied cattle consume more drinking water than smaller ovicaprids; as a result, cattle may be more directly reflecting precipitation water. Plants can vary in the depth at which they draw water and the relative amount of evapotranspiration^25^, which may result in variation in plant water δ^2^H, so that different plants eaten by cattle and ovicaprids may also be affecting the collagen δ^2^H values seen here. Additionally, differences in the amount of water ingested from plant matter (e.g. leaves) vs surface water may influence inter-species δ^2^H patterns. In using bone collagen δ^2^H and δ^18^O values for geo-location or palaeoenvironmental studies, caution must be used in selecting the species for comparison; δ^2^H in collagen shows clear differences between some animal species^26,27^.

Human bone collagen contains the least variation in both δ^2^H and δ^18^O across the Mediterranean. Although there is a difference in the δ^2^H of human bone collagen from the westernmost site in Spain compared with the more easterly sites, reflecting lower precipitation δ^2^H in the west (Fig. 2; Tables S2-S3), human bone collagen δ^18^O does not vary across the transect from Spain to Israel.

In contrast to the spread in δ^2^H values in collagen in cattle and ovicaprids (41 and 47‰ ranges, respectively), human bone collagen δ^2^H values from these sites cluster relatively tightly (27‰ range, Fig. 2). Similarly, δ^18^O values show higher ranges in the herbivores (5.4–5.5‰) than in the humans (3.2‰, Fig. 2). The reasons for this clustering in human collagen δ^2^H and δ^18^O values are likely due to human manipulation of food and environment, including agricultural practices (c.f. nitrogen isotopic changes^28,29^), animal management, and food preparation techniques. Cooking and boiling have been shown to increase δ^2^H and δ^18^O, for example^30,31^. Further work on the isotopic differences in human foods vs animal foods is warranted.

In the Mediterranean, hydrogen in bone collagen tracks precipitation δ^2^H values, especially so in cattle, while oxygen does not (Fig. 2, Table S3). In other studies, similarly, feather keratin also shows a weaker correlation of δ^18^O values with precipitation compared to δ^2^H values^32^. Across the Mediterranean region sampled here, from our westernmost to easternmost site, δ^18^O in precipitation increases by ~2‰ (−6.7‰ (site 1, west), −4.3‰ (sites 2–4, central), −4.8‰ (site 5, east), Table S1). This range is relatively small given the large range of δ^18^O values seen at a single site (e.g. 2.3–2.4‰ range in cattle at Los Berrocales and in Sardinia). This latter ‘biological noise’ may arise from variation in fractionation in δ^18^O values within a single species, perhaps due to food selection or physiological/biochemical parameters (c.f. different species-specific ‘calibration’ lines relating drinking water δ^18^O and biological apatite δ^18^O). In this case δ^18^O lacks geospatial discriminating power in bone collagen. Given the non-trivial analytical challenge in measuring collagen δ^18^O^16,33^, it is noteworthy that δ^2^H can be measured separately and more quickly with a Cr-packed reactor^34,35^, and that δ^2^H values in general have a larger range of variation relative to analytical uncertainty. In sum, the use of hydrogen isotopes is a more promising approach relative to δ^18^O for determination of migrants in the past.

## Conclusions

To our knowledge, we present here the first combined δ^2^H and δ^18^O values of collagen from different localities, from multiple species. These data demonstrate that δ^2^H in collagen varies with precipitation δ^2^H, most strongly in cattle, but also to a lesser extent in ovicaprids and humans. Hydrogen isotopes in bone collagen may be a powerful tool as a geographic discriminator over large geographic scales where the isotopic composition of meteoric water varies significantly. Given that δ^2^H can also vary between species, care should be taken when including and comparing different species^26,27^.

Humans demonstrate starkly different δ^2^H values from co-local fauna, and a smaller range in both δ^2^H and δ^18^O than in the fauna. We posit that human-specific dietary and cultural factors (e.g. cooking, agricultural modification) are at play, and the isotopic separation between humans and their habitat indicates a high degree of environmental manipulation.

## Methods

### Samples

Bone samples were obtained from five sites dated to the Bronze and Iron Ages: Los Berrocales (Spain, lat. 40.375, long. −3.578, 22–16^th^ century cal BCE)^36^, S’Urachi (Sardinia, lat. 40.015, long. 8.583, 10–5^th^ century cal BCE), Villamar (Sardinia, lat. 39.619, long. 8.96, 4–2^th^ century cal BCE), Monte Sirai (Sardinia, lat. 39.179, long. 8.488, 7–4^th^ century cal BCE), and Megiddo (Israel lat. 32.585, long. 35.184, 20–13^th^ century cal BCE, pers. comm. Melissa Cradic, Robert Homsher, Mario A.S. Martin). Site descriptions and the archaeological contexts of the samples are given in Supplementary Information. Bone fragments (human and faunal) were demineralized in 0.5 M EDTA, rinsed in distilled water numerous times, and freeze-dried.

### Mass spectrometry

Freeze-dried collagen samples (~300 μg) were weighed into silver capsules, and introduced via a zero-blank autosampler into a Thermal Conversion Elemental Analyzer (TCEA) for pyrolysis to H_2_ and CO gases. The gases were separated using a gas chromatograph (1.8 m length, 5 Å molecular sieve) and were introduced into an isotope-ratio mass spectrometer for δ^2^H and δ^18^O determination. δ^2^H and δ^18^O values were normalized on the VSMOW-SLAP scale, using VSMOW and SLAP in silver divots as references. The TCEA was packed with a Cr-metal powder filling, as described previously^34,35^, which has been shown to result in quantitative conversion of N-containing organics to H_2_ gas^34^. Consequently the δ^2^H data reported here is as obtained by the more newly-developed Cr-packing method. Our long-term reproducibility on organic samples is 3.6‰ for δ^2^H (Cr method) and 0.9‰ for δ^18^O.

We did not correct δ^2^H for exchangeable H, given that it is typically low in non-gelatinized collagen, all samples were analyzed in our laboratory over a few months, and there is still discordance between laboratories in how to perform water-collagen exchange experiments to carry out this correction (see ref. ^34^ for further discussion). It is worth noting that all bone collagen is “reset” with easily exchangeable hydrogen during the decalcification process. This does not affect our interpretation and conclusions.

We excluded putative diagenetically altered samples by the criteria described in ref. ^35^. Excluded samples had (one or more) of the following: lower mass fraction H, lower mass fraction O, or higher O/H ratios (Table S8). We also exclude from further analysis one sample from S’Urachi with a highly outlying radiocarbon date (Table S4), and juvenile (non-adult) humans from all sites (Table S7).

### Water isotope data

Water isotope data are from the Global Network of Isotopes in Precipitation (GNIP) of the International Atomic Energy Agency^37^. To approximate rainfall isotope values at each archaeological site, we used the data from two modern reporting sites near each archaeological site: Madrid-Retiro (lat. 40.41, long. −3.68) and Puerto de Navacerrada (40.79, −4.01) for Los Berrocales (Spain); Capo Caccia (40.57, 8.17) and Cagliari-Elmas (39.25, 9.07) for S’Urachi, Monte Sirai, Villamar (Sardinia, Italy); and Har Kna’an (32.97, 35.5) and Bet Dagan (32.00, 34.82) for Megiddo (Israel).

## Supplementary information

Supplementary Information.

## Data Availability

The datasets generated during and analyzed during the current study are available in Supplementary Information and are available from the corresponding author on reasonable request.
