# Supplementary material for: Mediterranean precipitation isoscape preserved in bone collagen δ2H
Source: Sci Rep. 2020 May 22;10:8579. doi: 10.1038/s41598-020-65407-0 (PMC7244594; doi:10.1038/s41598-020-65407-0)
Supplement: Supplementary file 1 — Supplementary Information. [file 41598_2020_65407_MOESM1_ESM.pdf]

Supplementary Information

Mediterranean precipitation isoscape preserved in bone collagen δ^2H

Linda M. Reynard, Saskia E. Ryan, Michele Guirguis, Miguel Contreras Martinez, Elisa Pompianu, Damià Ramis, Peter van Dommelen, Noreen Tuross

Modern and Past Precipitation in the Mediterranean Basin

The present precipitation in the Mediterranean has lower δ^2H and *d* excess values in the west and higher to the east (Table S1); *d* excess is significantly different between the sites (t tests pairwise by country, $p<3 \times 10^{-6}$), increasing from Spain to Sardinia to Israel. Greater aridity in the eastern Mediterranean (Israel in particular) results in higher *d* excess values and lower δ^2H vs $\delta^{18}O$ slopes.[1,2] At present at all sites considered here most precipitation falls in winter (December, January, February), and the discrepancy between winter and summer precipitation amounts increases from west to east (Table S1, note precipitation is 0 in summer at Megiddo (easternmost site)).

The past climate in the region is a matter of current research. It appears that the late Holocene (approx. 4000-2000 yr BP) saw the establishment of arid conditions, following a wetter early Holocene,[3-6] and a slightly colder mean annual temperature and winter temperature in the late Holocene compared to today.[5] Our sites all date to the later Holocene, after this shift in aridity. There is some spatial and temporal variability in the late Holocene precipitation trends. Precipitation anomalies (difference with present (1960-1990) or pre-industrial (100 BP)) are of the order of 6-8 mm/month for mean annual precipitation, with slightly higher magnitudes considering summer and winter individually. There are differences in patterns between summer/winter/annual precipitation anomalies at the sites considered here.[5,6] However, these differences in precipitation amount may not have had any consequence for the isotopic composition of rainfall. If the moisture sources and transport/rainout patterns were similar in the late Holocene to today, the general isotopic variation would be expected to be consistent with present values. Models suggest that the West African Monsoon, one candidate for summer moisture source in the past, did not reach the Mediterranean coast in the Holocene.[7]

Archaeological Context of Samples

Los Berrocales, Spain

The *Los Berrocales* (Madrid, Spain) archaeological site is a large settlement in the centre of the Iberian Peninsula occupied during the Middle Bronze Age (2000 – 1500 BCE). It consists of domestic spaces, tombs, storage pits and others subterranean structures and situated on a plain near the confluence of the Jarama and Manzanares rivers. The most characteristic elements of this site are the burials of 48 individuals in 40 excavated graves- single or multiple tombs with animal, ornamental or ceramic offerings. More details are given in refs. 8-10.

S'Urachi, Sardinia

The site of S'Urachi is situated at approximately 15 km from the central west coast of Sardinia, on the edge of a former wetland. A monumental multi-towered complex was built in the Bronze Age (later 2nd millennium BCE), while occupation during the 1st millennium was concentrated around the complex. All animal bone finds were part of a large deposit of domestic trash, which was dumped to backfill a 2-3 m deep ditch between the 9th and 5th centuries BCE. For further site details see refs. 11,12.

Villamar, Sardinia

The Punic site of Villamar is located in the lower Marmilla region of Sardinia, along an important passageway crossed by the Flumini Mannu (the Mannu River), which from the island's interior turns towards the middle Campidano plain. The settlement area, which has yielded evidence from the Copper Age, Bronze Age, Iron Age, and Punic period, is located below the centre of the modern village. The Punic cemetery was however never built over, because it is situated on a rocky outcrop. The research carried out so far has allowed us to define its use between the early fourth and early second century BCE. Various types of graves are documented, including hypogea with pit entrance and chamber or niche, trench, *alla cappuccina* and *enchytrismos* graves. The ritual of inhumation is used until the end of the third century BCE, when cremation became more prevalent. The remains analyzed in this paper come from Tomb 16, a rock-cut chamber tomb in use for over a century (late 4th-early 2nd century BCE) that accommodated the human remains and grave goods of 26 inhumations and two cremations. These include an unusually large number of fetuses and infants. In addition, several sheep or goats and dogs had also been deposited in the tomb. Further information is found in refs. 13-15.

Monte Sirai, Sardinia

The site of Monte Sirai (Carbonia) is a hill-top settlement located in south-western Sardinia, Italy. The site is comprised of three large sectors: the “acropolis” in the southern portion of the hill, the sanctuary “tophet” located in the northern portion of the site and the large necropolis located in the valley separating the settlement from the tophet. The necropolis was used between the end of the 7th century until the middle of the 4th century BCE and contained more than 360 tombs with a large variability in mortuary practices, including graves of single and multiple individuals, inhumations, primary cremation burials, *enchytrismoi* (transport amphorae as containers of child burials) and “semi-combustions”. The most intense period of use coincides with the Carthaginian hegemony in Sardinia, starting at the end of the 6th century BCE. Samples were obtained from a range of burial contexts excavated during 2005-2015 and dating from the early 6th to late 5th century BCE. Site information and context are found in refs. 16-19.

Megiddo, Israel

Megiddo is a tell site in the Jezreel Valley, Israel, which has been occupied from the late Neolithic (ca. 6000 BCE) through the Bronze and Iron Ages and to the mid/end of the first millennium BCE.[20-23] Excavations which have continued periodically from beginnings in the early 20th century have revealed habitation and fortification features.[20] Samples used in this study date from 20-13th century cal BCE (pers. comm. Melissa Cradic, Robert Homsher, Mario A.S. Martin).

Note about the samples from Sardinia
Permission for sampling of human remains from Monte Sirai and Villamar and of animal bones from S'Urachi was granted by the *Soprintendenza Archeologia Belle Arti e Paesaggio per la città metropolitana di Cagliari e le province di Oristano e sud Sardegna.* In particular, sampling permission was enabled by dott.ssa Sabrina Cisci for Monte Sirai (Carbonia, prot. #20587), dott.ssa Chiara Pilo for the Punic cemetery in Villamar (prot. #15667 and prot. #30918), and dott. Alessandro Usai for S'Urachi (San Vero Milis, prot. #15238). We thank the then director of the *soprintendenza*, arch. Fausto Martino, for authorizing the sampling, export and analysis, which made our research possible.

Supplementary Tables

Table S1 Precipitation amounts and isotopic values near site locations

	1, Los Berrocales		2, S'Urachi		3, Villamar & 4, Monte Sirai		5, Megiddo	
JJA precipitation (mm/month)	14.6	(2.7)[a]	7.5	(4.3)[a]	9.5	(3.9)[a]	0.0	(0)[a]
DJF precipitation (mm/month)	43.4	(6.8)[a]	73.3	(18.2)[a]	58.2	(17.8)[a]	140.0	(16.3)[a]
mean annual precip. (mm/month)	34.3	(2.8)[a]	48.2	(6.7)[a]	40.2	(6.4)[a]	50.3	(4.4)[a]
δ^2H mean (‰)	-45.2	(25.4)[b]	-23.5	(14.8)[b]	-23.5	(14.8)[b]	-19.5	(12.6)[b]
$\delta^{18}O$ mean (‰)	-6.7	(3.3)[b]	-4.3	(1.9)[b]	-4.3	(1.9)[b]	-4.8	(2.0)[b]
d excess mean (‰)	8.7	(7.4)[b]	12.4	(2.0)[b]	12.4	(2.0)[b]	17.9	(7.8)[b]

Precipitation amounts are from the Food and Agriculture Organization program New LocCLim, with data from 1961-1990, with interpolated values for each site.[24,25] Isotopic values are from the Global Network of Isotopes in Precipitation (IAEA).[26] Uncertainties are in parentheses, as follows: [a] standard error, propagated in quadrature from monthly values; [b] one standard deviation of the mean.

Table S2. Tukey's Honest Significant Differences and ANOVA p values between sites computed using R.[27] Bold values indicate non-significant differences.

	cattle δ^2H	ovicaprid δ^2H	human δ^2H	cattle $\delta^{18}O$	ovicaprid $\delta^{18}O$	human $\delta^{18}O$
Tukey HSD						
Los Berrocales (1) – Sardinia (2-4)	6.6×10^{-3}	**0.76**	1.6×10^{-5}	**0.81**	0.033	**0.12**
Sardinia (2-4) – Megiddo (5)	4×10^{-7}	4.6×10^{-3}	**0.58**	1.6×10^{-4}	7.7×10^{-4}	**0.61**
Los Berrocales (1) – Megiddo (5)	$< 10^{-7}$	2.9×10^{-3}	5.3×10^{-4}	1.2×10^{-3}	**0.46**	**0.094**
ANOVA	2.0×10^{-8}	1.5×10^{-3}	4.8×10^{-6}	1.9×10^{-4}	7.3×10^{-4}	**0.050**
sample numbers						
Los Berrocales (1)	6	8	19			
Sardinia (2-4)	10	15	26			
Megiddo (5)	4	9	5			

Los Berrocales (1) is the westernmost site; Sardinia (2-4) are central sites; Megiddo (5) is the easternmost site (see Fig. 1)

Table S3 Regression of collagen δ^2H and $\delta^{18}O$ vs precipitation δ^2H, $\delta^{18}O$, and *d*

		slope SE	intercept SE	r^2	p
cattle	$\delta^2H_{collagen}$= 3.4 *d* – 82.9 ‰	0.34	4.4	0.84	1.2×10^{-8}
(n=20)	$\delta^2H_{collagen}$= 0.75 δ^2H_{rain} – 19.4 ‰	0.19	6.0	0.46	0.001
	$\delta^{18}O_{collagen}$= 0.04 $\delta^{18}O_{rain}$ + 10.9 ‰	0.32	1.7	0.00	0.90
ovicaprids	$\delta^2H_{collagen}$= 1.5 *d* – 53.1 ‰	0.39	5.3	0.33	5.3×10^{-4}
(n=32)	$\delta^2H_{collagen}$= 0.35 δ^2H_{rain} – 23.5 ‰	0.15	4.4	0.15	0.026
	$\delta^{18}O_{collagen}$= -0.42 $\delta^{18}O_{rain}$ + 9.1 ‰	0.24	1.2	0.09	0.09
humans	$\delta^2H_{collagen}$= 1.4 *d* – 11.8 ‰	0.29	3.4	0.33	1.2×10^{-5}
(n=50)	$\delta^2H_{collagen}$= 0.39 δ^2H_{rain} + 16.9 ‰	0.07	2.3	0.40	7.0×10^{-7}
	$\delta^{18}O_{collagen}$= 0.20 $\delta^{18}O_{rain}$ + 11.1 ‰	0.09	0.5	0.09	0.03

SE = standard error

Table S4 Cattle bone collagen isotopic values and mass fractions

sample name	site[a]	$\delta^{18}O$ (‰)	sd[b]	$\delta^{2}H$[c] (‰)	sd[b]	% H[c]	% O[d]	O/H[e]	C/N[f]
06/46/ESP/1166/5	1, Los Berrocales	9.1		-47	4.0	6.8	30.2	5.0	3.17
06/46/ESP/1763/8 B	1, Los Berrocales	9.4		-57	1.8	6.0	26.7	5.0	3.22
06/46/ESP/356/10	1, Los Berrocales	11.3	0.4	-52	2.8	5.9	27.6	5.4	3.39
06/46/ESP/383/31	1, Los Berrocales	11.3	1.1	-54	0.6	6.4	26.3	4.8	3.18
06/46/ESP/503/18	1, Los Berrocales	10.6		-51	0.0	6.2	26.0	5.0	3.26
06/46/ESP/595/52	1, Los Berrocales	10.4		-47	2.3	6.0	27.3	5.1	3.25
SU 62 A E	2, S'Urachi	10.9		-45	2.2	6.2	28.6	5.1	3.15
SU 56 D E	2, S'Urachi	11.1		-34	2.8	6.3	26.6	4.6	3.22
SU 60 C E	2, S'Urachi	9.8		-47	2.9	6.4	27.8	4.9	3.34
SU 66 A E	2, S'Urachi	10.4		-47	3.2	6.2	26.8	4.8	3.28
SU 59 F E	2, S'Urachi	10.2	0.3	-45	2.4	6.3	27.2	4.8	3.27
SU 54 A E	2, S'Urachi	9.7		-42	4.2	6.1	28.3	5.1	3.23
SU 72 D E	2, S'Urachi	9.6		-41		6.3	25.9	4.9	3.16
SU 30 C E	2, S'Urachi	8.7		-50	1.8	6.6	28.5	4.8	3.24
SU 53_E	2, S'Urachi	9.4		-44	0.4	6.4	28.5	4.9	3.16
SU 12 A E	2, S'Urachi	10.6		-37	0.9	6.7	29.6	5.0	3.21
SU 56 C E[g]	*2, S'Urachi*	*8.8*		*-43*	*1.3*	*6.1*	*28.8*	*5.0*	*3.30*
2016/H/063 PT008 013/2 (Meg C 3)	5, Megiddo	13.4		-16	1.0	5.8	24.6	5.1	3.13
2016/H/062 PT030 044/2 (Meg C 6)	5, Megiddo	13.5		-21	0.8	5.6	27.2	5.1	3.15
2016/H/064 PT005 18 (Meg C 7)	5, Megiddo	11.0		-26	2.7	6.1	28.2	4.7	3.17
2016/H/062 PT002 008/1 (Meg C 8)	5, Megiddo	14.2		-19	3.0	5.7	26.3	4.9	3.18

[a] site ages: Los Berrocales: 22-16th century cal BCE, S'Urachi: 10-5th century cal BCE, Megiddo: 20-13th century cal BCE; [b] 1 standard deviation of replicates; [c] measured by Cr-packed reactor [28,29] with sucrose used as a mass fraction reference; [d] measured by glassy C-packed reactor [28,29] with sucrose used as a mass fraction reference; [e] O/H by mass fraction, measured by glassy C-packed reactor [28,29] with sucrose used as mass fraction reference; [f] atomic C/N (used by convention); [g] excluded from further analysis due to an outlying radiocarbon date

Table S5 Ovicaprid bone collagen isotopic values and mass fractions

sample name	site[a]	$\delta^{18}O$ (‰)	sd[b]	$\delta^{2}H$[c] (‰)	sd[b]	% H[c]	% O[d]	O/H[e]	C/N[f]
06/46/ESP/1793/27	1, Los Berrocales	11.3	0.7	-25	0.8	6.6	28.9	5.0	3.22
06/46/ESP/1793/55	1, Los Berrocales	14.2		-30	2.5	6.2	27.8	4.9	3.19
06/46/ESP/1771/19	1, Los Berrocales	12.5	0.5	-30	5.2	6.6	26.1	4.8	3.14
06/46/ESP/1793/46-47	1, Los Berrocales	12.6	0.2	-40	1.0	6.4	26.0	4.8	3.20
06/46/ESP/277/28	1, Los Berrocales	10.0	0.0	-59	3.8	6.2	25.8	4.7	3.20
06/46/ESP/358/25	1, Los Berrocales	8.9	0.0	-43	0.6	6.7	28.3	5.1	3.24
06/46/ESP/503/36	1, Los Berrocales	11.6	0.1	-43	1.0	6.4	30.5	4.9	3.20
06/46/ESP/405/41	1, Los Berrocales	12.4		-35	1.5	6.7	27.7	5.0	3.16
SU 62 B E	2, S'Urachi	11.4		-31	0.9	6.4	27.4	4.9	3.18
SU 71 A E	2, S'Urachi	9.1		-38	1.6	6.5	29.0	4.9	3.22
SU 56 A E	2, S'Urachi	10.8		-42	1.9	6.2	25.6	4.9	3.17
SU 57 B E	2, S'Urachi	11.1		-34	2.9	6.3	26.1	4.8	3.34
SU 60 A E	2, S'Urachi	11.9		-32	1.2	6.5	27.7	4.8	3.19
SU 59 A E	2, S'Urachi	9.4		-36	2.9	6.7	27.8	4.8	3.18
SU 59 E E	2, S'Urachi	11.4	0.1	-37	2.5	6.7	31.2	5.1	3.26
SU 54 B E	2, S'Urachi	9.7		-38	4.0	6.4	27.4	5.1	3.22
SU 30 B E	2, S'Urachi	8.8	0.1	-36	4.0	6.4	27.4	4.8	3.18
SU 30 D E	2, S'Urachi	10.7	0.0	-26			24.5	4.8	3.15
SU 31 A E	2, S'Urachi	10.8		-34	2.9	6.6	26.9	4.8	3.20
SU 59 D D	2, S'Urachi	10.4		-50	0.2	6.3	27.0	4.8	3.27
319 CAP A	3, Villamar	10.1		-37	0.8	7.0	28.6	4.9	3.23
319 CAP B	3, Villamar	9.9		-32	0.0	7.1	28.4	4.8	3.19
319 CAP C	3, Villamar	10.3		-35	1.8	6.9	28.2	4.9	3.21
2016/H/045 PT006 LB103 29 (Meg 29)	5, Megiddo	12.6		-27	3.9	6.0	26.1	4.9	3.17
2016/H/045 PT008 LB203 26 (Meg 26)	5, Megiddo	12.0		-27	1.1	5.9	27.1	5.1	3.13
2016/H/062 PT019 LB075 2 (Meg 2)	5, Megiddo	11.4	0.2	-28	0.7	6.3	30.2	5.0	3.17

Table S5 cont'd

sample name	site[a]	$\delta^{18}O$ (‰)	sd[b]	$\delta^{2}H$[c] (‰)	sd[b]	% H[c]	% O[d]	O/H[e]	C/N[f]
2016/H/062 PT019 LB075 3 (Meg 3)	5, Megiddo	12.4		-27	1.3	5.8	26.7	5.1	3.22
2016/H/062 PT020 LB084 5 (Meg 5)	5, Megiddo	12.2		-28	4.3	6.2	26.1	5.0	3.16
2016/H/062 PT020 LB084 6 (Meg 6)	5, Megiddo	11.4		-34	0.2	6.0	25.7	5.0	3.14
2016/H/063 PT004 LB058 24 (Meg 24)	5, Megiddo	14.1		-11	1.3	6.2	26.4	4.9	3.16
2016/H/063 PT008 LB109 18 (Meg 18)	5, Megiddo	11.9	0.4	-28	3.0	6.2	30.6	5.1	3.16
2016/H/064 PT003 LB037 19 (Meg 19	5, Megiddo	13.2		-12	1.9	6.2	26.8	4.9	3.13

[a] site ages: Los Berrocales: 22-16th century cal BCE, S'Urachi: 10-5th century cal BCE, Villamar: 4-2th century cal BCE, Megiddo: 20-13th century cal BCE; [b] 1 standard deviation of replicates; [c] measured by Cr-packed reactor [28,29] with sucrose used as a mass fraction reference; [d] measured by glassy C-packed reactor [28,29] with sucrose used as a mass fraction reference; [e] O/H by mass fraction, measured by glassy C-packed reactor [28,29] with sucrose used as mass fraction reference; [f] atomic C/N (used by convention)

Table S6 Adult human bone collagen isotopic values and mass fractions

sample name		site[a]	$\delta^{18}O$ (‰)	sd[b]	$\delta^{2}H$[c] (‰)	sd[b]	% H[c]	% O[d]	O/H[e]	C/N[f]
ESP 10.1	06/46/ESP/1162/19	1, Los Berrocales	10.1		-9	0.5	6.2	31.8	5.1	3.15
ESP 11	06/46/ESP/1176/12	1, Los Berrocales	10.2	0.1	0	3.1	6.3	25.9	4.7	3.18
ESP 12	06/46/ESP/1186/4	1, Los Berrocales	10.8		1	0.6	6.9	28.3	5.0	3.16
ESP 13.1	06/46/ESP/1212/8	1, Los Berrocales	9.0	0.2	-6	1.0	6.6	27.2	4.9	3.15
ESP 15	06/46/ESP/1246/2	1, Los Berrocales	10.2		2	0.9	6.7	27.2	4.9	3.16
ESP 16	06/46/ESP/1261/5	1, Los Berrocales	8.9	0.2	-11	0.0	6.8	29.3	5.0	3.24
ESP 17	06/46/ESP/1306/5	1, Los Berrocales	10.8	0.0	-5	1.8	6.4	26.9	4.8	3.25
ESP 18	06/46/ESP/1327/14	1, Los Berrocales	9.5	0.3	-5	0.5	6.3	25.8	4.8	3.15
ESP 2	06/46/ESP/250/1	1, Los Berrocales	9.4	0.0	6	0.8	6.6	27.5	5.0	3.20
ESP 20	06/46/ESP/1424/1	1, Los Berrocales	9.6	0.2	5	5.6	7.0	26.7	4.8	3.12
ESP 21.1	06/46/ESP/1456/17	1, Los Berrocales	9.4		-4	0.7	6.4	26.1	4.6	3.17
ESP 22	06/46/ESP/1771/17	1, Los Berrocales	9.0		-2	0.7	7.0	30.1	5.0	3.26
ESP 23	06/46/ESP/1794/6	1, Los Berrocales	9.3		2	4.0	6.5	25.5	4.5	3.18
ESP 24	06/46/ESP/1803/21	1, Los Berrocales	9.1		6	0.2	6.8	27.3	4.9	3.19
ESP 26	06/46/ESP/1934/9	1, Los Berrocales	9.8	0.1	2	0.1	6.4	26.2	4.8	3.17
ESP 27	06/46/ESP/1944/14	1, Los Berrocales	8.7		-11	2.4	6.1	27.3	4. 9	3.15
ESP 28	06/46/ESP/1946/2	1, Los Berrocales	10.4	0.3	5	5.3	6.6	24.2	4.8	3.16
ESP 3	06/46/ESP/304/5	1, Los Berrocales	9.8		4	0.5	6.5	29.6	5.0	3.17
ESP 4	06/46/ESP/401/15	1, Los Berrocales	9.5	0.2	3	1.0	6.3	27.2	4.8	3.17
321 CR3		3, Villamar	8.7	0.0	5	1.6	6.5	30.6	5.2	3.27
322		3, Villamar	9.2	0.1	11	0.9	6.8	28.3	4.9	3.24
327 CR2		3, Villamar	9.7	0.5	0	0.8	6.6	26.8	5.0	3.21
324 CR2		3, Villamar	9.2	0.3	11	1.6	7.0	27.4	4.9	3.19
324 INV 209		3, Villamar	9.5	0.1	1	3.6	6.7	27.6	4.9	3.23
327 CR4		3, Villamar	9.5	0.1	16	3.5	6.7	26.8	4.8	3.23
327 CR3		3, Villamar	8.8	0.9	12	4.6	6.8	28.0	4.9	3.22

Table S6 cont'd

sample name		site[a]	$\delta^{18}O$ (‰)	sd[b]	$\delta^{2}H$[c] (‰)	sd[b]	% H[c]	% O[d]	O/H[e]	C/N[f]
324 CR3		3, Villamar	9.8	0.2	17	3.0	6.8	28.1	5.1	3.24
323 CR1		3, Villamar	9.2	0.1	8	2.0	6.3	27.1	4.9	3.22
MS 320		4, Monte Sirai	11.0	0.7	2	1.3	6.5	30.7	5.2	3.25
MS 284		4, Monte Sirai	9.1	0.4	12	1.0	5.7	25.2	5.1	3.23
MS 296		4, Monte Sirai	10.8		5	1.2	6.6	24.3	5.0	3.23
MS 345		4, Monte Sirai	10.7	0.4	4	0.9	6.3	25.5	4.7	3.36
MS 321		4, Monte Sirai	9.9	0.5	-1	4.0	6.2	26.7	4.9	3.23
MS 354		4, Monte Sirai	11.1	0.2	5	0.1	6.1	27.1	4.9	3.18
MS 3 SEP I		4, Monte Sirai	10.8	0	10	1.3	6.5	26.2	4.9	3.29
MS 334		4, Monte Sirai	10.3	0.5	1	5.3	6.1	26.5	4.9	3.29
MS 3 SEP II		4, Monte Sirai	9.7	0.1	9	1.8	6.2	26.4	4.7	3.19
MS 5		4, Monte Sirai	10.5		2	0.5	6.0	25.4	4.5	3.30
MS 347		4, Monte Sirai	9.8	0.2	6	6.3	6.4	27.3	4.7	3.26
MS 256		4, Monte Sirai	11.1	0.1	13	2.6	5.8	26.4	5.1	3.27
MS 237		4, Monte Sirai	10.9	0.1	16	0.3	6.6	30.7	5.2	3.26
MS 6		4, Monte Sirai	10.0	0.4	11	0.6	6.4	25.9	5.0	3.28
MS 285		4, Monte Sirai	11.6		5	0.2	6.2	25.4	4.9	3.19
MS 281		4, Monte Sirai	11.0	0.1	13	0.1	6.2	29.1	5.1	3.25
MS 348		4, Monte Sirai	10.8	0.1	3	0.1	6.1	25.8	4.8	3.24
S10092	2006/J/037 PT018 LB024	5, Megiddo	11.9		5	0.6	5.7	25.5	5.2	3.23
S10093	2006/J/037 PT022 LB032	5, Megiddo	10.5	0.1	13	0.3	6.3	26.3	4.9	3.22
S10098	2012/K/027 LB006	5, Megiddo	9.9		8	2.8	5.9	26.9	5.0	3.21
H191-H192	2016/H/062 PT023 113/192 – 115/191	5, Megiddo	10.5		10		5.8	26.4	4.9	3.09
H146	2016/H/063 PT005 086/146	5, Megiddo	9.5	0.1	15	1.3	6.2	26.6	4.9	3.10

[a] site ages: Los Berrocales: 22-16th century cal BCE, Villamar: 4-2th century cal BCE, Monte Sirai: 7-4th century cal BCE, Megiddo: 20-13th century cal BCE; [b] 1 standard deviation of replicates; [c] measured by Cr-packed reactor[28,29] with sucrose used as a mass fraction reference; [d] measured by glassy C-packed reactor [28,29] with sucrose used as a mass fraction reference; [e] O/H by mass fraction, measured by glassy C-packed reactor[28,29] with sucrose used as mass fraction reference; [f] atomic C/N (used by convention)

Table S7 Sub-adult human bone collagen isotopic values and mass fractions

sample name		age[a]	site[b]	$\delta^{18}O$ (‰)	sd[c]	$\delta^{2}H^{d}$ (‰)	sd[c]	% H[d]	% O[e]	O/H[f]	C/N[g]
ESP 10.2	06/46/ESP/1162/20	child-1	1, Los Berrocales	9.5		-4	0.9	6.8	30.8	4.9	3.20
ESP 21.2	06/46/ESP/1456/18	child-2	1, Los Berrocales	10.5	0.1	7	1.5	6.9	27.1	5.0	3.23
ESP 5	06/46/ESP/435/5	child-2	1, Los Berrocales	10.6		3	0.3	6.2	26.0	4.9	3.15
ESP 6	06/46/ESP/609/30	child-1	1, Los Berrocales	9.5	0.1	-6	1.4	5.9	25.1	4.8	3.19
ESP 7	06/46/ESP/801/22	child-2	1, Los Berrocales	9.2		-9	0.5	5.7	26.7	5.0	3.19
ESP 8	06/46/ESP/830/10	child-2	1, Los Berrocales	9.9	0.1	2	1.1	6.4	25.7	4.7	3.17
ESP 9	06/46/ESP/966/7	child-2	1, Los Berrocales	10.8	0.5	7	0.4	7.1	28.8	4.9	3.18
320 IDI		child	3, Villamar	9.6	0.2	8	3.1	6.8	27.8	4.8	3.26
327 ID3		child	3, Villamar	9.7	0.1	3	0.1	6.8	26.9	4.9	3.23
319 CR2		child	3, Villamar	10.0		3	1.8	6.7	28.6	5.0	3.27
S10104	2010/J/172 PT002 LB002	unknown	5, Megiddo	8.4		7	0.5	6.4	31.1	5.0	3.21
S10264	2010/K/088 PT002 LB002	child-1	5, Megiddo	9.8	0.0	5	1.7	6.6	27.7	4.9	3.20
S10096	2010/K/106 LB002	child-1	5, Megiddo	10.2		-9	7.5	6.6	28.6	5.0	3.24
S10097	2010/K/106 LB004	infant	5, Megiddo	10.4	0.1	19	4.9	6.4	27.0	5.0	3.20
S10267	2012/K/113 PT001 LB001	infant	5, Megiddo	10.8	0.2	20	3.2	6.2	26.9	4.8	3.22
S10268	2014/K/105 PT006 LB025	infant	5, Megiddo	9.6	0.3	-1	4.9	6.5	26.8	4.7	3.21
S10100	2014/K/119 PT012 LB031	child-2	5, Megiddo	10.5		9	2.0	6.4	30.8	5.0	3.21
S4519-S4520	2014/K/159 PT001 LB003	child-1	5, Megiddo	10.4		6		5.8	26.1	4.9	3.21
S10101	2014/K/175 PT004 LB007	infant	5, Megiddo	9.1	0.0	-6		6.1	24.8	4.7	3.21
Meg H75-H76	2016/H/064 PT003 041/76 – 014/75	child-2	5, Megiddo	10.6		14		6.1	27.3	5.0	3.12

[a] Age groups: 'infant' <2 y; 'child-1' 2-8 y; 'child-2' 8-17 y; 'child' sub-adult, no further information; [b] site ages: Los Berrocales: 22-16th century cal BCE, Villamar: 4-2th century cal BCE, Megiddo: 20-13th century cal BCE; [c] 1 standard deviation of replicates; [d] measured by Cr-packed reactor [28,29] with sucrose used as a mass fraction reference; [e] measured by glassy C-packed reactor [28,29] with sucrose used as a mass fraction reference; [f] O/H by mass fraction, measured by glassy C-packed reactor [28,29] with sucrose used as mass fraction reference; [g] atomic C/N (used by convention)

Table S8 Bone collagen mass fractions of diagenetically altered and excluded samples

sample name		site[a]	species	% H[b]	% O[c]	O/H[d]	C/N[e]
SU 63 B E		2, S'Urachi	cattle	3.8	21.8	6.8	3.24
SU 57 A E		2, S'Urachi	ovicaprid	4.9	28.4	5.7	3.19
MS 230		4, Monte Sirai	human	2.1	12.4	9.7	3.34
MS 241		4, Monte Sirai	human	0.5	5.4	15.7	
MS 242		4, Monte Sirai	human	5.8	29.7	5.6	3.23
MS 329		4, Monte Sirai	human	5.6	21.2	5.8	3.26
Meg C 1	2016/H/062 PT002 008/2	5, Megiddo	cattle	5.5	22.8	5.7	3.12
Meg C 2	2016/H/063 PT001 001	5, Megiddo	cattle	5.0	29.1	7.0	3.28
Meg C 4	2016/H/063 PT008 013/1	5, Megiddo	cattle	5.6	28.7	5.6	3.17
Meg C 5	2016/H/062 PT030 44/1	5, Megiddo	cattle	4.8	26.7	5.9	3.20
Meg C 9	2016/H/063 PT001 002	5, Megiddo	cattle	4.6	25.7	6.3	3.21
Meg 21	2016/H/065 PT011 LB118 21	5, Megiddo	ovicaprid	5.5	24.7	5.3	3.13
Meg 27	2016/H/045 PT007 LB143 27	5, Megiddo	ovicaprid	5.9	27.5	5.6	3.12
Meg 28	2016/H/045 PT005 LB088 28	5, Megiddo	ovicaprid	4.2	24.9	5.3	3.18
S10266	2012/K/096 PT002 LB002	5, Megiddo	human	4.9	23.2	5.1	3.26
S10270	2014/K/175 PT003 LB005	5, Megiddo	human	5.0	22.7	5.0	3.20
S4517	1998/K/100 PT006 LB007	5, Megiddo	human	2.7	16.8	4.9	3.19
S4518	2006/M/056 PT003	5, Megiddo	human	4.6	17.2	5.2	3.19

[a] site ages: S'Urachi: 10-5th century cal BCE, Monte Sirai: 7-4th century cal BCE, Megiddo: 20-13th century cal BCE; [b] measured by Cr-packed reactor [28,29] with sucrose used as a mass fraction reference; [c] measured by glassy C-packed reactor [28,29] with sucrose used as a mass fraction reference; [d] O/H by mass fraction, measured by glassy C-packed reactor [28,29] with sucrose used as mass fraction reference; [e] atomic C/N (used by convention)

References

1. Sharp, Z. *Principles of stable isotope geochemistry.* 2nd ed. doi: 10.5072/FK2GB24S9F (2017).

2. Gat, J. R. & Carmi, I. Evolution of the isotopic composition of atmospheric waters in the Mediterranean Sea area. *J. Geophys. Res*. **75,** 3039-3048 (1970).

3. Finné, M., Holmgren, K., Sundqvist, H. S., Weiberg, E. & Lindblom, M. Climate in the eastern Mediterranean, and adjacent regions, during the past 6000 years -a review. *J. Archaeol. Sci*. **38,** 3153-3173 (2011).

4. Roberts, N., Brayshaw, D., Kuzucuoğlu, C., Perez, R. & Sadori, L. The mid-Holocene climatic transition in the Mediterranean: causes and consequences. *The Holocene* **21,** 3-13 (2011).

5. Mauri, A., Davis, B. A. S., Collins, P. M. & Kaplan, J. O. The climate of Europe during the holocene: a gridded pollen-based reconstruction and its multi-proxy evaluation. *Quat. Sci. Rev.* **112,** 109-127 (2015).

6. Peyron, O. et al. Precipitation changes in the Mediterranean basin during the Holocene from terrestrial and marine pollen records: a model-data comparison. *Clim. Past* **13,** 249-265 (2017).

7. Brayshaw, D. J., Rambeau, C. M. C. & Smith, S. J. Changes in Mediterranean climate during the Holocene: insights from global and regional climate modelling. *The Holocene* **21**, 15-31 (2011).

8. Aliaga, R & Megías, M. Los Berrocales (Madrid): un yacimiento de la Edad de Bronce en la confluencia Jarama-Henares. *Patrimonio Arqueológico de Madrid*/8. Universidad Autónoma de Madrid – Arqueomedia (2011).

9. Barroso, R., Ramírez, P., González Martín, A., Candelas González, N., Rojas, J. & López-Sáez, J. A. Enterramientos de la Edad del Bronce en la Meseta Sur a partir del Sector 22, Yuncos (Toledo). *Munibe (Antropologia-Arkeologia)* **65**, 117-136 (2014).

10. Pérez Villa, A. *Prácticas funerarias de la Edad del Bronce en la cuenca medio-alta del Tajo*. Tésis Doctoral. Universidad Nacional de Educacion a Distancia (España, 2014).

11. Stiglitz, A., Díes Cusí, E., Ramis, D., Roppa, A. & van Dommelen, P. Intorno al nuraghe: notizie preliminari sul Progetto S'Urachi (San Vero Milis, OR). *Quaderni della Soprintendenza Archeologica per le province di Cagliari e Oristano* **26**, 191-218 (2015).

12. van Dommelen, P., Díes Cusí, E., Gosner, L., Hayne, J., Pérez Jordà, G., Ramis, D., Roppa, A. & Stiglitz, A. Un millennio di storie: nuove notizie preliminari sul progetto S'Urachi (San Vero Milis, OR), 2016-2018, *Quaderni della Soprintendenza Archeologia, Belle Arti e Paesaggio per la città metropolitana di Cagliari e le province di Oristano e Sud Sardegna* **29**, 141-166 (2018).

13. Piga, C., Piroddi, L., Pompianu, E., Ranieri, G., Stocco, S. & Trogu, A. Integrated Geophysical and Aerial Sensing Methods for Archaeology: A Case History in the Punic Site of Villamar (Sardinia, Italy). *Remote Sens*. **6**, 10986-11012 (2014).

14. Pompianu E. Nuovi scavi nella necropoli punica di Villamar (2013-2015). *Fasti Online*. **395**, 1-28. http://www.fastionline.org/docs/FOLDER-it-2017-395.pdf (2017).

15. Pompianu E. Cartagine in Sardegna. Nota su alcuni contesti con incinerazioni dalla necropoli di Villamar. *Folia Phoenicia* **3**, 99-116 (2019).

16. Guirguis, M. Necropoli fenicia e punica di Monte Sirai- Indagini archeologiche 2005-2007. *Studi di Storia antica e di Archeologia* **7** (2010).

17. Guirguis, M., Murgia, C. & Pla Orquín, R. Archeoantropologia e bioarcheologia nella necropoli di Monte Sirai (Carbonia-Italia). Risultati delle analisi su alcuni contesti della prima età punica (fine VI-inizi IV sec. a.C.) in *From the Mediterranean to the Atlantic: People, Goods and Ideas between East and West. 8th International Congress of Phoenician and Punic Studies, Italy-Sardinia, Carbonia-Sant'Antioco 21th-26th October 2013* (ed. Guirguis, M). Folia Phoenicia, 1, Fabrizio Serra Editore, vol. I, 282-299 (2017).

18. Guirguis, M., Pla Orquín, R. & Pompianu, E. Premature Deaths in Punic Sardinia. The Perception of Childhood in Funerary Contexts from Monte Sirai and Villamar in *From Invisible to Visible. New Methods and Data for the Archaeology of Infant and Child Burials in Pre-Roman Italy and Beyond* (ed. Tabolli, J.) Studies in Mediterranean Archaeology, CXLIX, 207-215 (2018).

19. Piga, G. Guirguis, M. & Allué, E. Funerary rituals and ideologies in the Phoenician-Punic necropolis of Monte Sirai (Carbonia, Sardinia, Italy) in *The Archaeology of Cremation. Burned human remains in funerary studies* (ed. Thompson, T.) Studies in Funerary Archaeology, 8, Oxbow Books, 97-122 (2015).

20. Finkelstein, I. & Ussishkin, D. Back to Megiddo. *Biblical Archaeol. Rev.* **20** (1994).

21. Finkelstein et al. 2006; Finkelstein, I., Ussishkin, B. and Halpern, B. (eds.). 2006. Megiddo IV: The 1998-2002 Seasons. Tel Aviv. Institute of Archaeology, Tel Aviv University (2006).

22. Finkelstein, I., Ussishkin, D. & Cline, E. H. Megiddo V: The 2004-2008 Seasons. Institute of Archaeology, Tel Aviv University (2013).

23. Toffolo, M. B., Arie, E., Martin, M. A. S., Boaretto, E. & Finkelstein, I. Absolute chronology of Megiddo, Israel, in the Late Bronze and Iron Ages: high-resolution radiocarbon dating. *Radiocarbon* **56**, 221–244 (2014).

24. Food and Agriculture Organization of the United Nations. *New LocCLim*. v.1.10. Accessible at: http://www.fao.org/land-water/land/land-governance/land-resources-planning-toolbox/category/details/en/c/1032167 (2006).

25. Grieser, J., Gommes, R. & Bernardi, M. New LocClim - the local climate estimator of FAO. *Geophys. Res. Abstr*. **8,** 08305 (2006).

26. IAEA/WMO. Global Network of Isotopes in Precipitation. The GNIP Database. Accessible at: http://www.iaea.org/water (2019).

27. R Core Team. R: A language and environment for statistical computing. https://www.R-project.org (2017).

28. Reynard, L. M. & Tuross, N. Hydrogen isotopic analysis with a chromium-packed reactor of organic compounds of relevance to ecological, archaeological, and forensic applications. *Rapid Commun. Mass Spectrom*. **30,** 1857-1864 (2016).

29. Reynard L. M., Ryan S. E. & Tuross N. The interconversion of δ^2H values of collagen between thermal conversion reactor configurations. *Rapid Commun. Mass Spectrom*. **33,** 678-682 (2019).
